# Supplementary material for: MEK inhibitors reduce cellular expression of ACE2, pERK, pRb while stimulating NK-mediated cytotoxicity and attenuating inflammatory cytokines relevant to SARS-CoV-2 infection
Source: Oncotarget. 2020 Nov 17;11(46):4201–23. doi: 10.18632/oncotarget.27799 (PMC7679035; doi:10.18632/oncotarget.27799)
Supplement: Supplementary file 1 [file oncotarget-11-4201-s001.pdf]

# MEK inhibitors reduce cellular expression of ACE2, pERK, pRb while stimulating NK-mediated cytotoxicity and attenuating inflammatory cytokines relevant to SARS-CoV-2 infection

## SUPPLEMENTARY MATERIALS

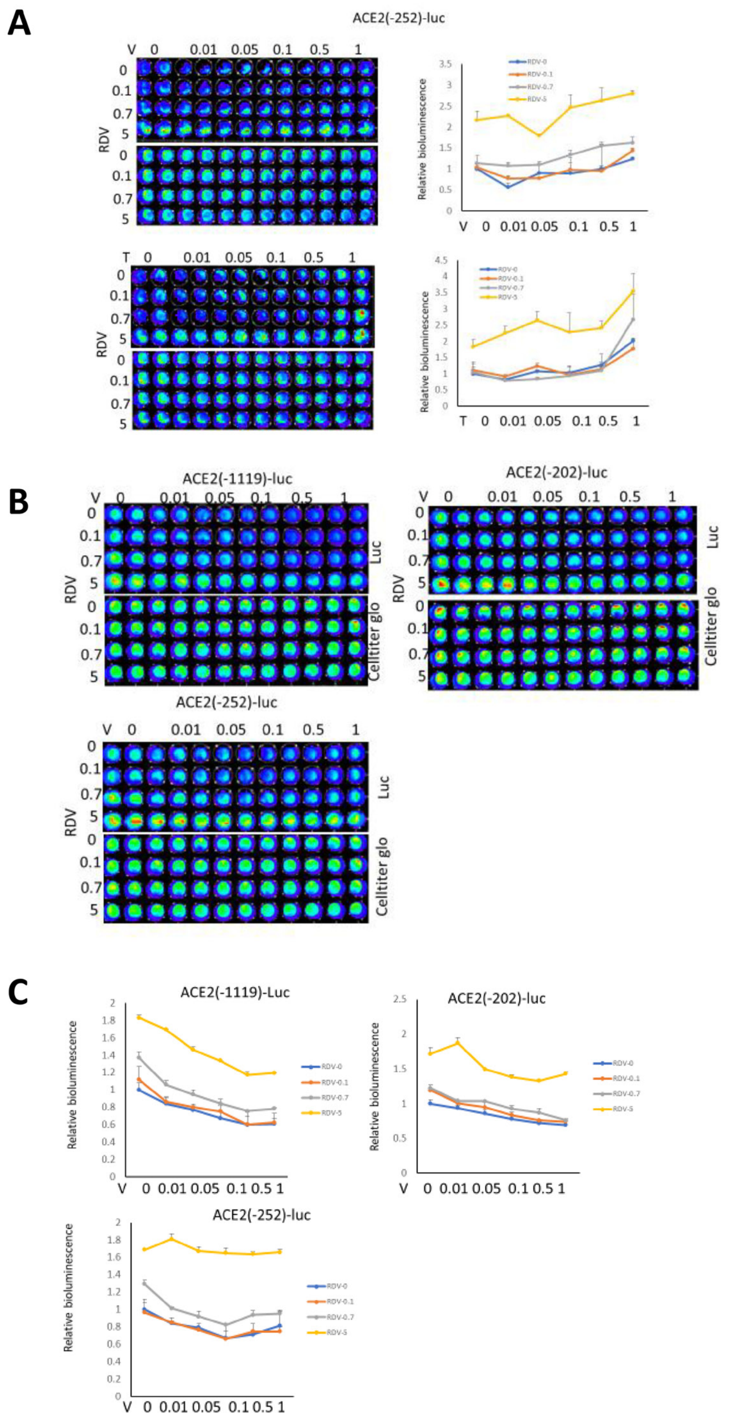

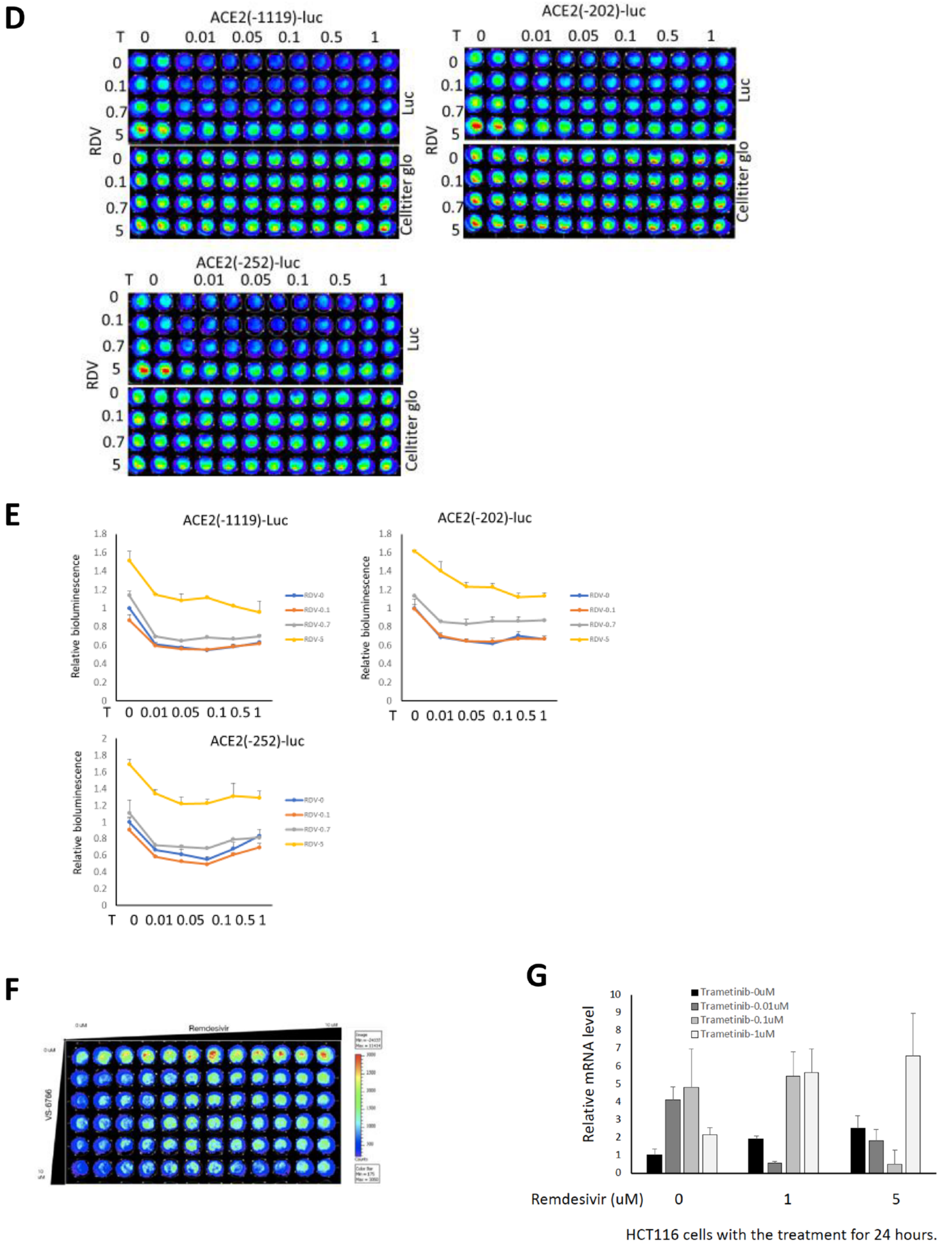


**Supplementary Figure 1: Modulation of multiple different ACE2-promoter luciferase reporters and ACE2 mRNA expression by remdesivir and MEKi in different cell lines.** (**A**) ACE2(-252) luc reporter assay in Calu-6 cells treated with remdesivir and VS-6766 (upper panel), or remedisivir and trametinib (lower panel). (**B**) ACE2- luc reporter assay in HCT116 cells treated with remdesivir and VS-6766 for 24 hours. (**C**) The relative bioluminescence value in (B). (**D**) ACE2- luc reporter assay in HCT116 cells treated with remdesivir and trametinib for 24 hours. (**E**) The relative bioluminescence value in (D). (**F**) ACE2(-1119)-Luc reporter assay in HCT116 cells treated with remdesivir and VS6766. The data from the luciferase reporter assays (A–F) was normalized to cell viability and plotted relative to cells with treated with DMSO as a control. Data are expressed as mean ± SD. V, VS-6766 (μM). RDV, Remdesivir (μM). T, Trametinib (μM). (**G**) ACE2 mRNA level in HCT116 cells treated with remdesivir and trametinib for 24 hours. mRNA levels were quantified by qRT-PCR. Data were normalized to GAPDH expression and plotted relative to cells treated with DMSO as a control. Data are expressed as mean ± SD.

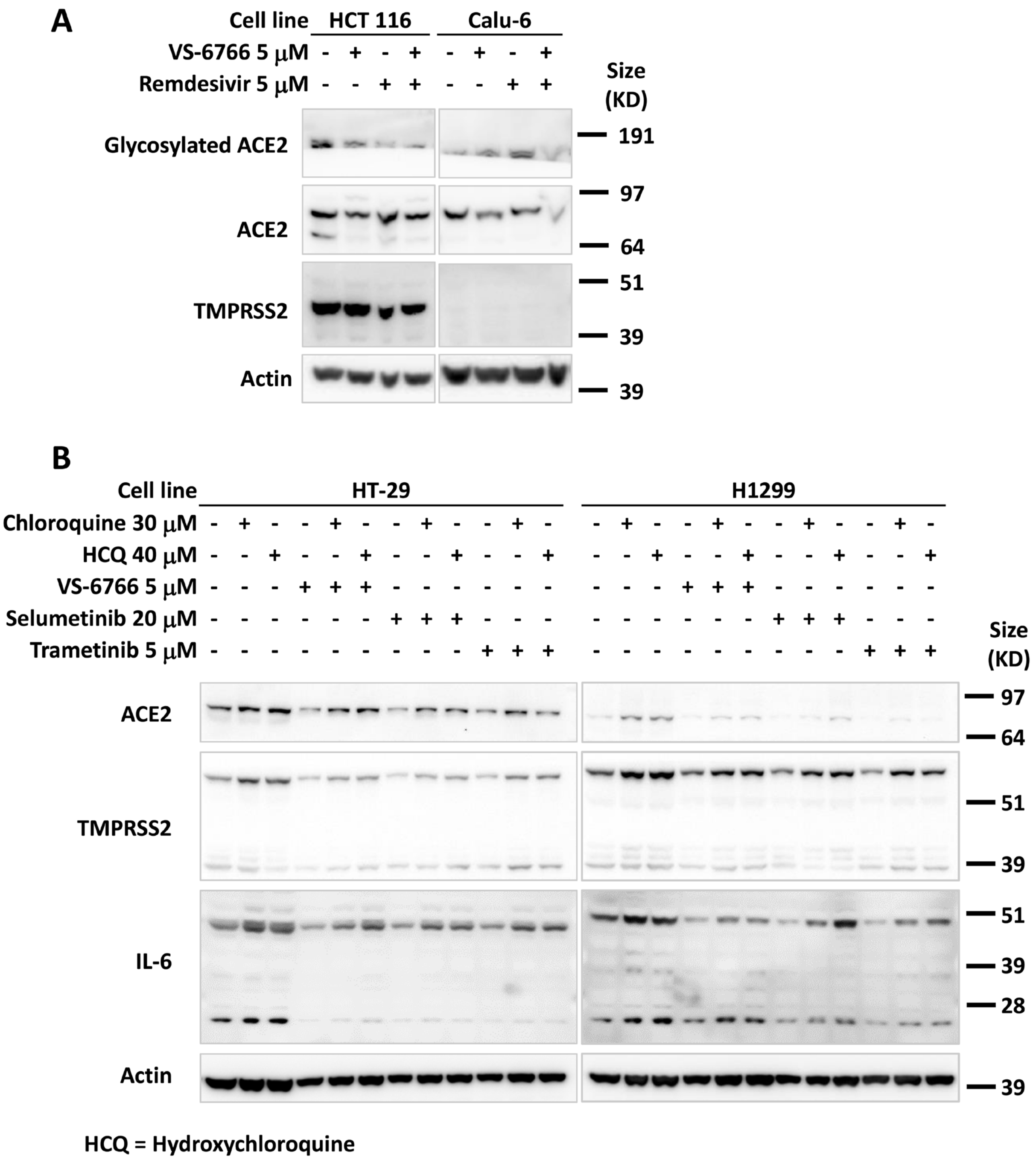


**Supplementary Figure 2: Effect of remdesivir and VS-6766 on ACE2 and TMPRSS2 in human colorectal and NSCLC cells.** (**A**) HCT116 human CRC cells and Calu-6 human NSCLC cells were treated with remdesivir and VS-6766 at the indicated doses for 48 hours. Glycosylated ACE2, ACE2 and TMPRSS2 (full-length and Serine Protease-domain) were probed with cell signaling 4355, Abnova PAB13444, and Sigma MABF2158 antibodies. α-Actin was probed with Sigma A5441 antibody as a loading control. (**B**) H1299 human NSCLC cells were treated with chloroquine, hdroxychloroquine and MEK inhibitors VS-6766, selumetinib, and trametinib at the indicated doses for 48 hours. Glycosylated ACE2, ACE2 and TMPRSS2 (full-length and Serine Protease-domain) were probed with cell signaling 4355, Abnova PAB13444, and Sigma MABF2158 antibodies. α-Actin was probed with Sigma A5441 antibody as a loading control.

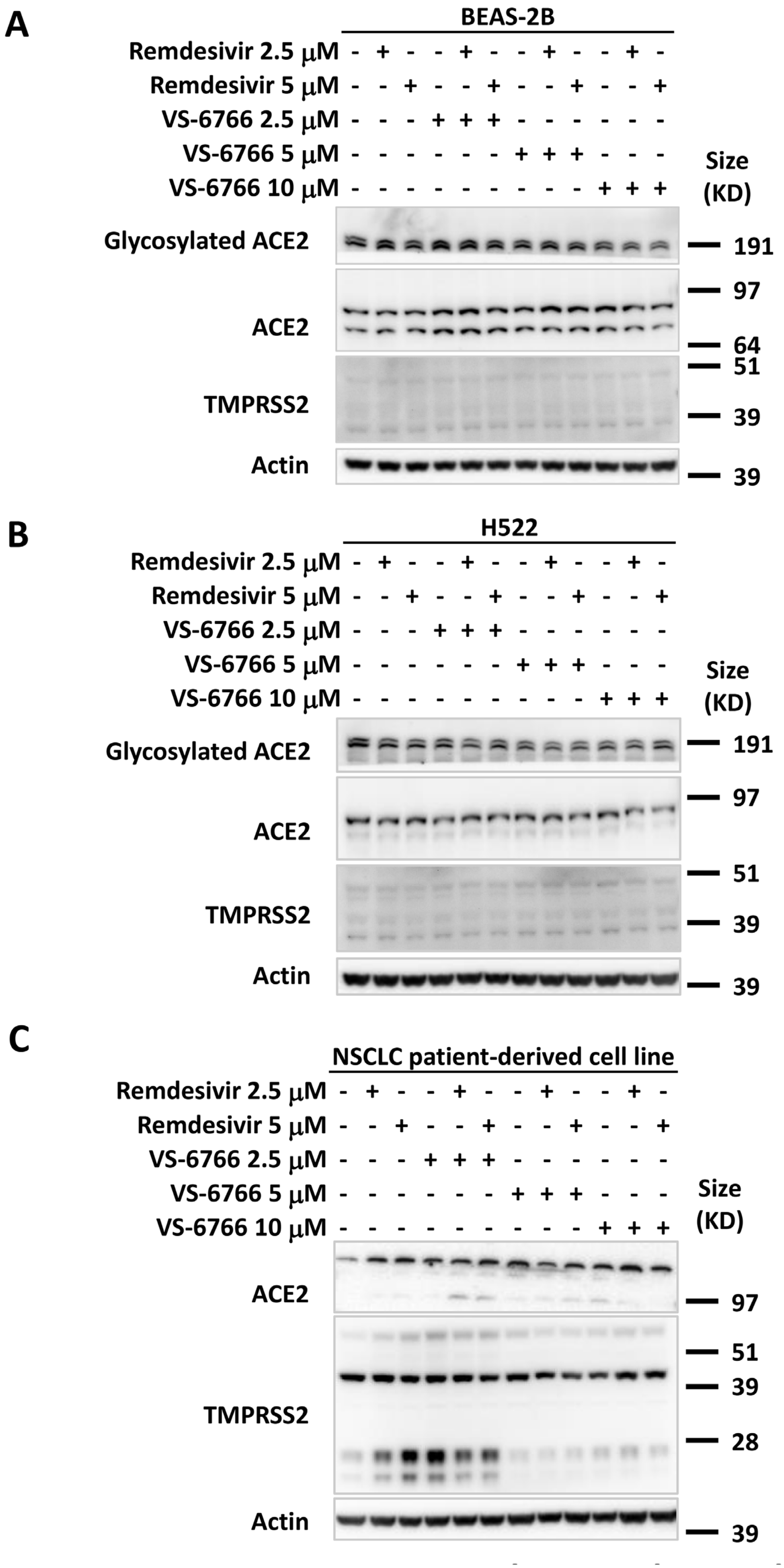
A
BEAS-2B
Remdesivir 2.5 μM - + - - + - - + - - + -
Remdesivir 5 μM - - + - - + - - + - - +
VS-6766 2.5 μM - - - + + + - - - - - -
VS-6766 5 μM - - - - - - + + + - - -
VS-6766 10 μM - - - - - - - - - + + +
Size (KD)
Glycosylated ACE2
191
ACE2
97
64
TMPRSS2
51
39
Actin
39
B
H522
Remdesivir 2.5 μM - + - - + - - + - - + -
Remdesivir 5 μM - - + - - + - - + - - +
VS-6766 2.5 μM - - - + + + - - - - - -
VS-6766 5 μM - - - - - - + + + - - -
VS-6766 10 μM - - - - - - - - - + + +
Size (KD)
Glycosylated ACE2
191
ACE2
97
TMPRSS2
51
39
Actin
39
C
NSCLC patient-derived cell line
Remdesivir 2.5 μM - + - - + - - + - - + -
Remdesivir 5 μM - - + - - + - - + - - +
VS-6766 2.5 μM - - - + + + - - - - - -
VS-6766 5 μM - - - - - - + + + - - -
VS-6766 10 μM - - - - - - - - - + + +
Size (KD)
ACE2
97
TMPRSS2
51
39
28
Actin
39

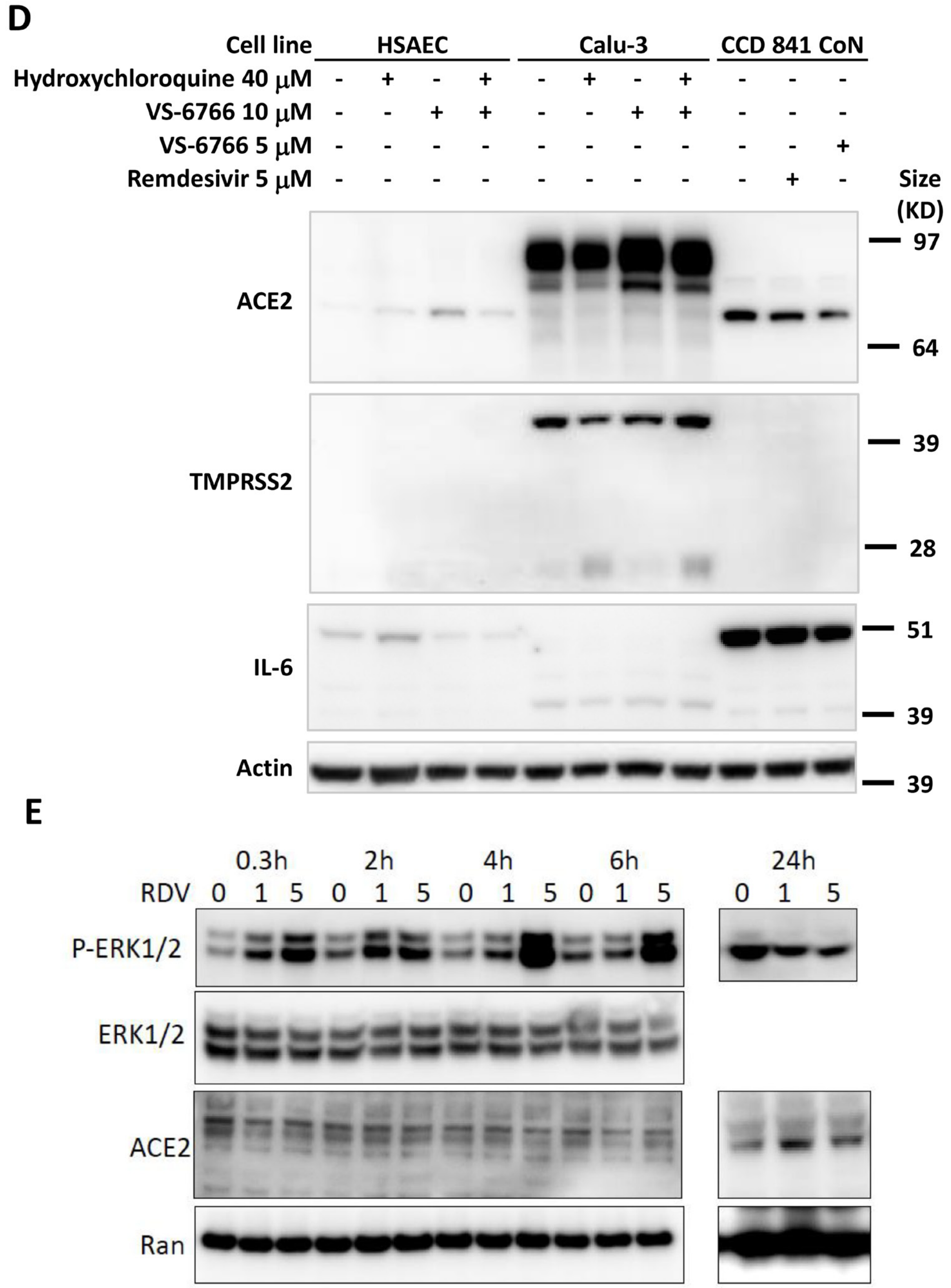


**Supplementary Figure 3: Effects of remdesivir, VS-6766 and Hydroxychloroquine on ACE2, TMPRSS2 and IL-6 in human lung and colon cells and expression of ACE2 and TMPRSS2 in cells of GI tract origin.** (**A**) BEAS-2B normal human bronchial airway epithelial cells were treated with remdesivir and VS-6766 at the indicated doses for 48 hours. Glycosylated ACE2, ACE2 and TMPRSS2 (full-length and Serine Protease-domain) were probed with cell signaling 4355, Abnova PAB13444, and Sigma MABF2158 antibodies. α-Actin was probed with Sigma A5441 antibody as a loading control. (**B**) H522 human NSCLC cells were treated with remdesivir and VS-6766 at the indicated doses for 48 hours. Glycosylated ACE2, ACE2 and TMPRSS2 (full-length and Serine Protease-domain) were probed with cell signaling 4355, Abnova PAB13444, and Sigma MABF2158 antibodies. α-Actin was probed with Sigma A5441 antibody as a loading control. (**C**) NSCLC patient-derived cell line was treated with remdesivir and VS-6766 at the indicated doses for 24 hours. ACE2 and TMPRSS2 (full-length and Serine Protease-domain) were probed with Santa Cruz sc-390851 and Sigma MABF2158 antibodies. α-Actin was probed with Sigma A5441 antibody as a loading control. (**D**) Human Primary Small Airway Normal Epithelial Cells (HSAEC) and Calu-3 human NSCLC cells (Both are type II alveolar cells) were treated with hydroxychloroquine and VS-6766 at the indicated doses for 24 hours. (**E**) Calu-6 cells were treated with remdesivir at 0, 1, or 5 μM for 0.3, 2, 4, 6, or 24 hr as indicated. Western blots were performed to immunoblot for expression of pERK1/2, ERK1/2, ACE2, or Ran (loading control) as indicated.

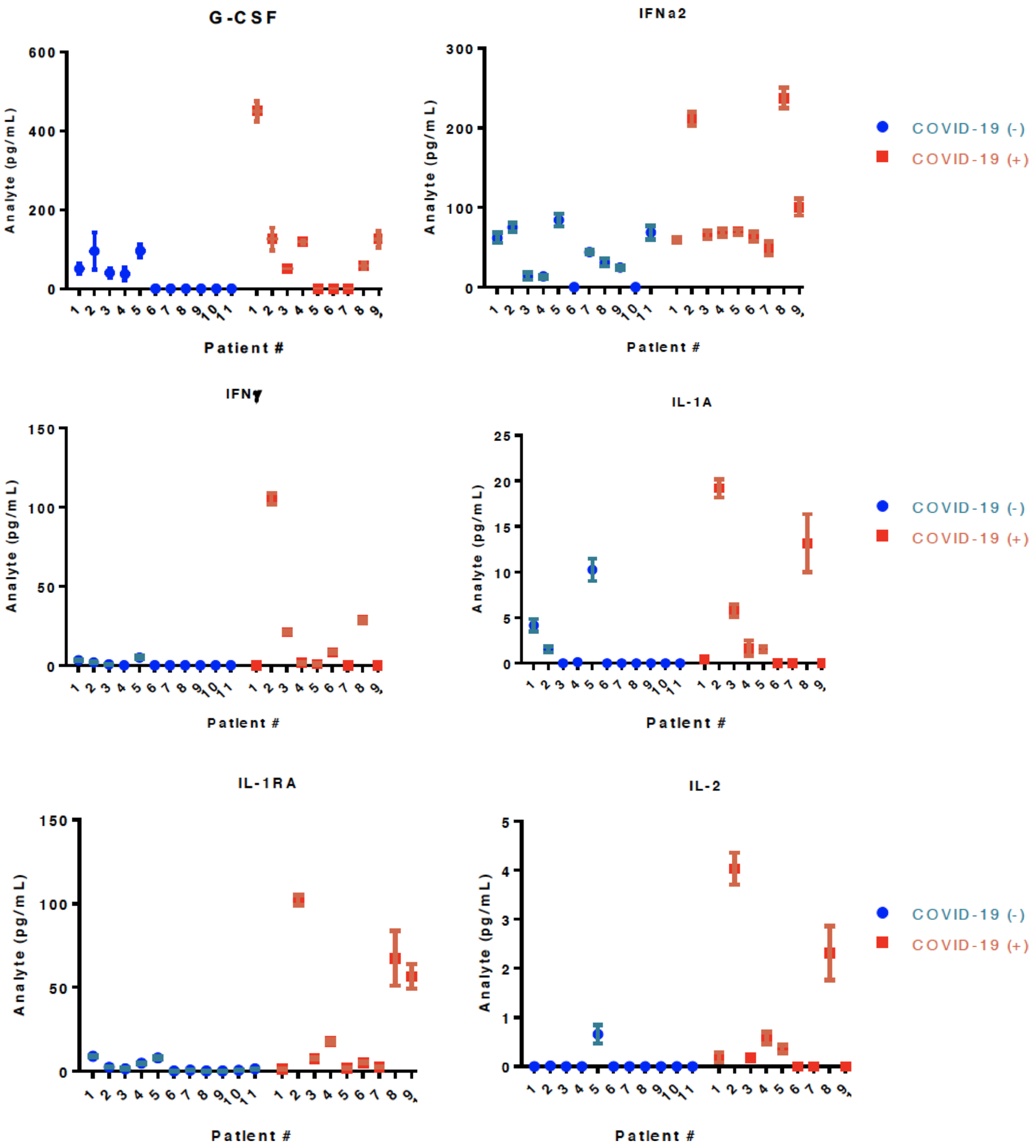
G-CSF
IFNa2
IFNγ
IL-1A
IL-1RA
IL-2
Analyte (pg/mL)
Patient #
COVID-19 (-)
COVID-19 (+)

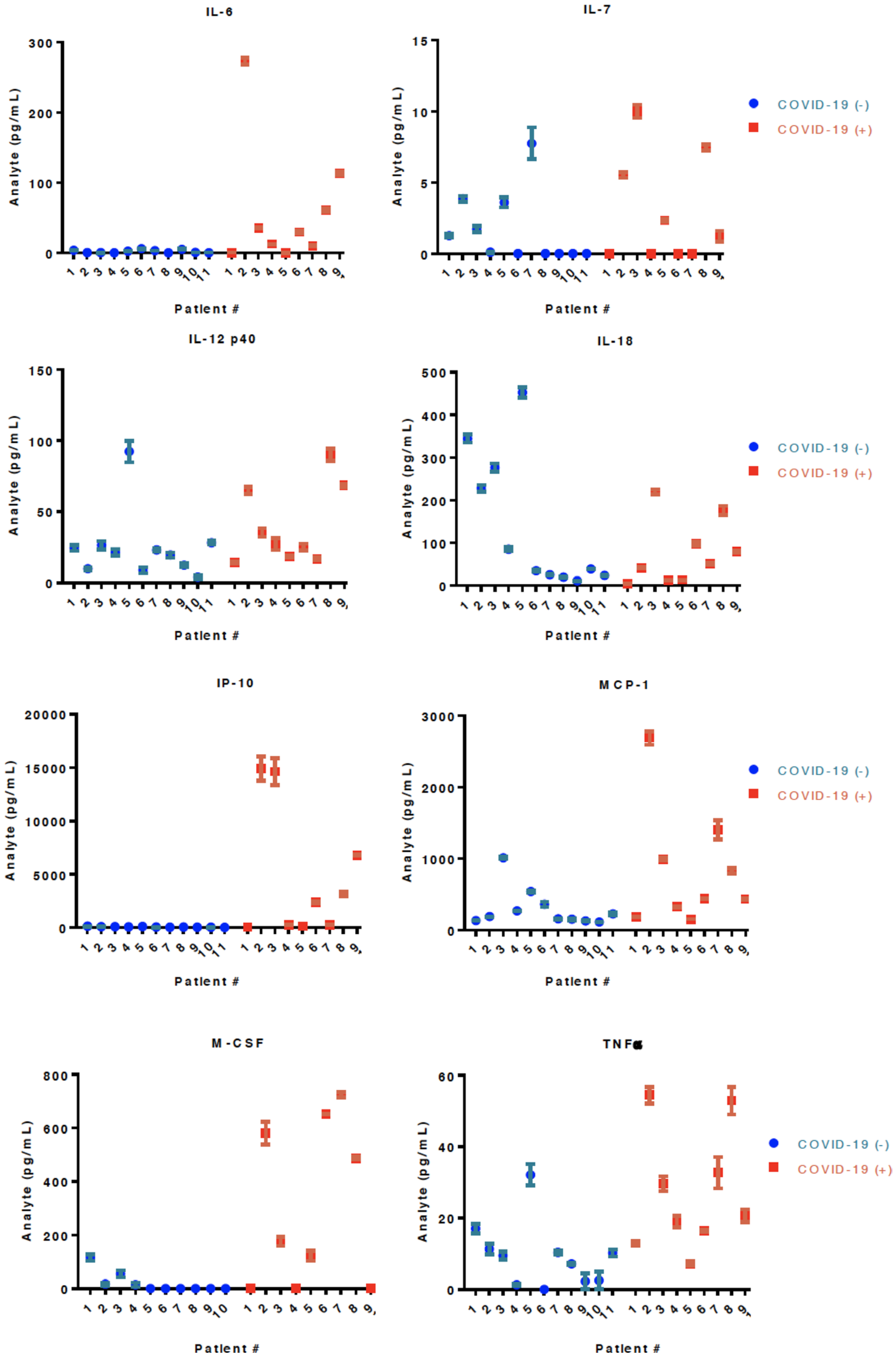


**Supplementary Figure 4: Pilot study of cytokine array profiles in COVID-19-(+) patient plasma versus control patient plasma.** Cytokine levels detected in plasma are shown for individual normal ($N = 11$) or COVID-19-(+) ($N = 9$) patients. For the COVID-19-(+) patients the patient numbers (1-9) correspond sequentially with the numbers listed in Tables 1–5. For the COVID-19-(+) patients the cytokine levels are shown for each patient in Table 5.

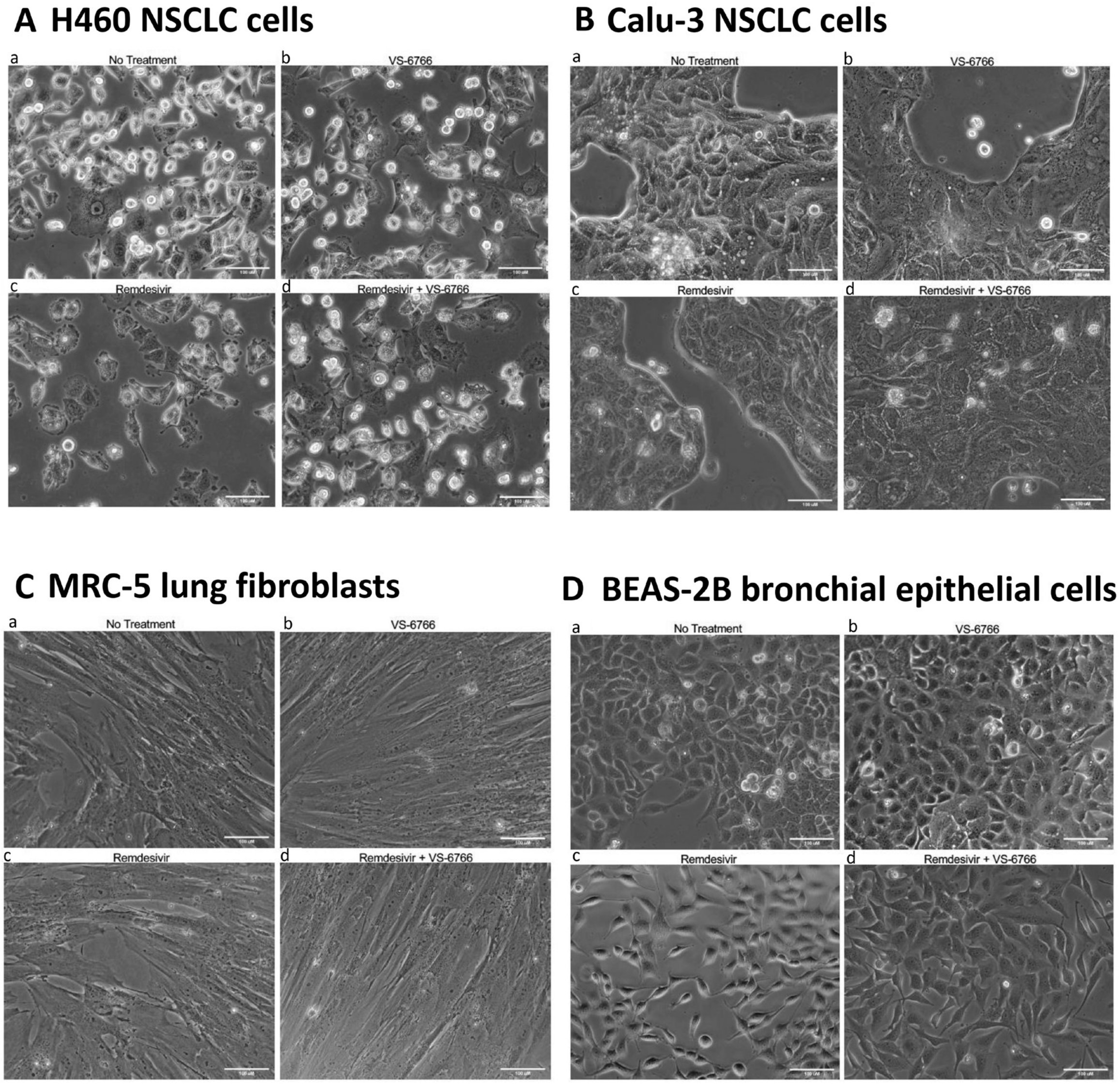


**Supplementary Figure 5:** No morphological changes were observed following drug treatment with VS-6766, remdesivir or the combination in (**A**) H460 or (**B**) Calu-3 lung cancer cells, (**C**) normal lung fibroblasts (MRC-5) or (**D**) bronchial airway epithelial cells (BEAS-2B). Cells were treated for 48 hours with the following drugs in the sub-panels: (a) control; (b) 10 µM VS-6766; (c) 10 µM remdesivir; and (d) a combination treatment of 10 µM Remdesivir and 10 µM VS-6766. Micrographs were taken after 48 hours of treatment. Scale bar represents 100 µm.

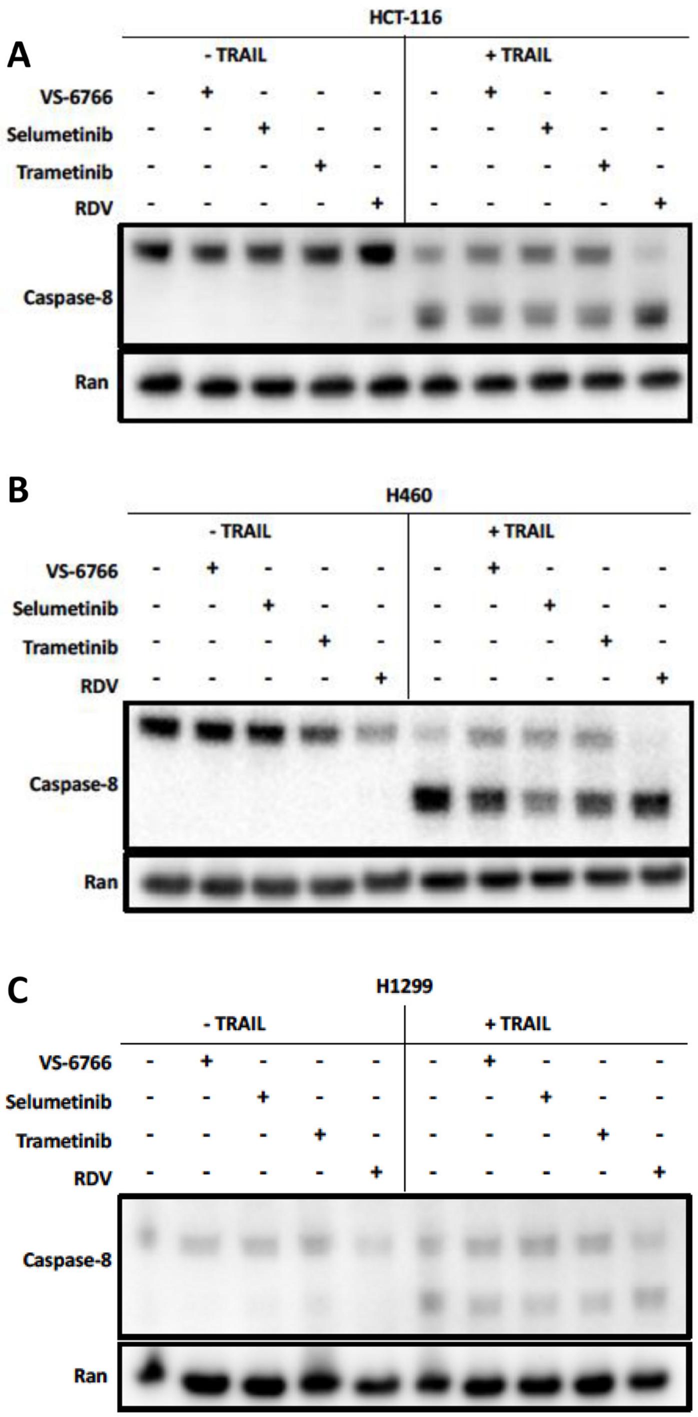


**Supplementary Figure 6: MEK inhibitors or remdesivir do not inhibit TRAIL-mediated apoptosis.** Effects of VS-6766 (5 µM), selumetinib (10 µM), trametinib (5 µM), or remdesivir (5 µM) treatment for 24 hours alone or in combination with TRAIL (50 ng/mL) for 4 additional hours on cleaved caspase 8 in HCT116 colorectal cancer (**A**), H460 (**B**) or H1299 (**C**) lung cancer cells.

**A**

| | | 1 | 2 | 3 | 4 | 5 | 6 | 7 | 8 | 9 | 10 | 11 | 12 |
|---|---|---|---|---|---|---|---|---|---|---|---|---|---|
| A | Chloroquine | 0 | 0 | 0.78 | 1.56 | 3.125 | 6.25 | 12.5 | 25 | 50 | 100 | 200 | 400 |
| B | Chloroquine | 0 | 0 | 0.78 | 1.56 | 3.125 | 6.25 | 12.5 | 25 | 50 | 100 | 200 | 400 |
| C | Hydroxchloroquine | 0 | 0 | 0.49 | 0.78 | 1.56 | 3.125 | 6.25 | 12.5 | 25 | 50 | 100 | 200 |
| D | VS-6766 | 0 | 0 | 0.08 | 0.16 | 0.31 | 0.63 | 1.25 | 2.5 | 5 | 10 | 20 | 40 |
| E | Selumetinib | 0 | 0 | 0.08 | 0.16 | 0.31 | 0.63 | 1.25 | 2.5 | 5 | 10 | 20 | 40 |
| F | Trametinib | 0 | 0 | 0.08 | 0.16 | 0.31 | 0.63 | 1.25 | 2.5 | 5 | 10 | 20 | 40 |
| G | U0126 | 0 | 0 | 0.08 | 0.16 | 0.31 | 0.63 | 1.25 | 2.5 | 5 | 10 | 20 | 40 |
| H | U0126 | 0 | 0 | 0.08 | 0.16 | 0.31 | 0.63 | 1.25 | 2.5 | 5 | 10 | 20 | 40 |

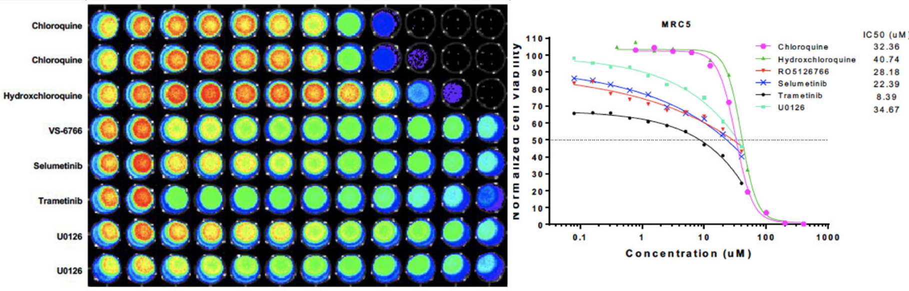


**B**

| | | 1 | 2 | 3 | 4 | 5 | 6 | 7 | 8 | 9 | 10 | 11 | 12 |
|---|---|---|---|---|---|---|---|---|---|---|---|---|---|
| A | Chloroquine | 0 | 0 | 0.78 | 1.56 | 3.125 | 6.25 | 12.5 | 25 | 50 | 100 | 200 | 400 |
| B | Chloroquine | 0 | 0 | 0.78 | 1.56 | 3.125 | 6.25 | 12.5 | 25 | 50 | 100 | 200 | 400 |
| C | Hydroxchloroquine | 0 | 0 | 0.49 | 0.78 | 1.56 | 3.125 | 6.25 | 12.5 | 25 | 50 | 100 | 200 |
| D | VS-6766 | 0 | 0 | 0.08 | 0.16 | 0.31 | 0.63 | 1.25 | 2.5 | 5 | 10 | 20 | 40 |
| E | Selumetinib | 0 | 0 | 0.08 | 0.16 | 0.31 | 0.63 | 1.25 | 2.5 | 5 | 10 | 20 | 40 |
| F | Trametinib | 0 | 0 | 0.08 | 0.16 | 0.31 | 0.63 | 1.25 | 2.5 | 5 | 10 | 20 | 40 |
| G | U0126 | 0 | 0 | 0.08 | 0.16 | 0.31 | 0.63 | 1.25 | 2.5 | 5 | 10 | 20 | 40 |
| H | U0126 | 0 | 0 | 0.08 | 0.16 | 0.31 | 0.63 | 1.25 | 2.5 | 5 | 10 | 20 | 40 |

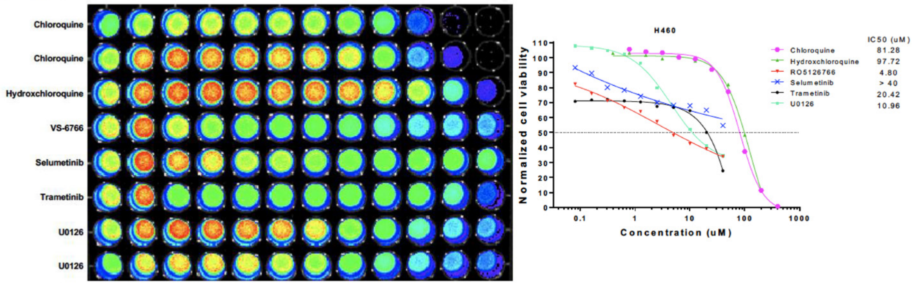


**C**

| | | 1 | 2 | 3 | 4 | 5 | 6 | 7 | 8 | 9 | 10 | 11 | 12 |
|---|---|---|---|---|---|---|---|---|---|---|---|---|---|
| A | Chloroquine | 0 | 0 | 0.78 | 1.56 | 3.125 | 6.25 | 12.5 | 25 | 50 | 100 | 200 | 400 |
| B | Chloroquine | 0 | 0 | 0.78 | 1.56 | 3.125 | 6.25 | 12.5 | 25 | 50 | 100 | 200 | 400 |
| C | Hydroxchloroquine | 0 | 0 | 0.49 | 0.78 | 1.56 | 3.125 | 6.25 | 12.5 | 25 | 50 | 100 | 200 |
| D | VS-6766 | 0 | 0 | 0.08 | 0.16 | 0.31 | 0.63 | 1.25 | 2.5 | 5 | 10 | 20 | 40 |
| E | Selumetinib | 0 | 0 | 0.08 | 0.16 | 0.31 | 0.63 | 1.25 | 2.5 | 5 | 10 | 20 | 40 |
| F | Trametinib | 0 | 0 | 0.08 | 0.16 | 0.31 | 0.63 | 1.25 | 2.5 | 5 | 10 | 20 | 40 |
| G | U0126 | 0 | 0 | 0.08 | 0.16 | 0.31 | 0.63 | 1.25 | 2.5 | 5 | 10 | 20 | 40 |
| H | U0126 | 0 | 0 | 0.08 | 0.16 | 0.31 | 0.63 | 1.25 | 2.5 | 5 | 10 | 20 | 40 |

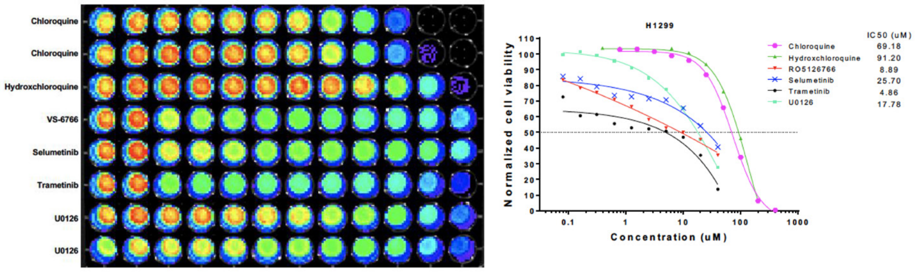


**D**

| | | 1 | 2 | 3 | 4 | 5 | 6 | 7 | 8 | 9 | 10 | 11 | 12 |
|---|---|---|---|---|---|---|---|---|---|---|---|---|---|
| A | Chloroquine | 0 | 0 | 0.78 | 1.56 | 3.125 | 6.25 | 12.5 | 25 | 50 | 100 | 200 | 400 |
| B | Chloroquine | 0 | 0 | 0.78 | 1.56 | 3.125 | 6.25 | 12.5 | 25 | 50 | 100 | 200 | 400 |
| C | Hydroxchloroquine | 0 | 0 | 0.49 | 0.78 | 1.56 | 3.125 | 6.25 | 12.5 | 25 | 50 | 100 | 200 |
| D | VS-6766 | 0 | 0 | 0.08 | 0.16 | 0.31 | 0.63 | 1.25 | 2.5 | 5 | 10 | 20 | 40 |
| E | Selumetinib | 0 | 0 | 0.08 | 0.16 | 0.31 | 0.63 | 1.25 | 2.5 | 5 | 10 | 20 | 40 |
| F | Trametinib | 0 | 0 | 0.08 | 0.16 | 0.31 | 0.63 | 1.25 | 2.5 | 5 | 10 | 20 | 40 |
| G | U0126 | 0 | 0 | 0.08 | 0.16 | 0.31 | 0.63 | 1.25 | 2.5 | 5 | 10 | 20 | 40 |
| H | U0126 | 0 | 0 | 0.08 | 0.16 | 0.31 | 0.63 | 1.25 | 2.5 | 5 | 10 | 20 | 40 |

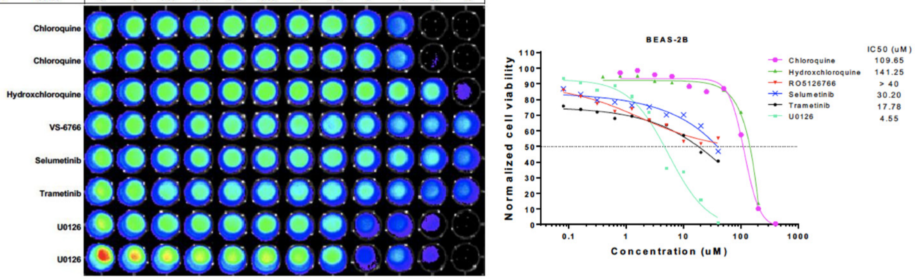

**Supplementary Figure 7:** Cell viability assay of MRC-5 (**A**), H460 (**B**), H1299 (**C**) and BEAS-2B (**D**) treated with Chloroquine, Hydroxychloroquine and MEK inhibitors (VS-6766, Selumetinib, Trametinib and U0126). MRC-5 normal human lung fibroblast cells, H460 and H1299 human NSCLC cells, and BEAS-2B normal human bronchial airway epithelial cells were treated as indicated for 72 hours and CellTiter-Glo was added to acquire cell viability images with the IVIS system. GraphPad Prism 6 was used to plot the dose-response curve and calculate the IC50 values.

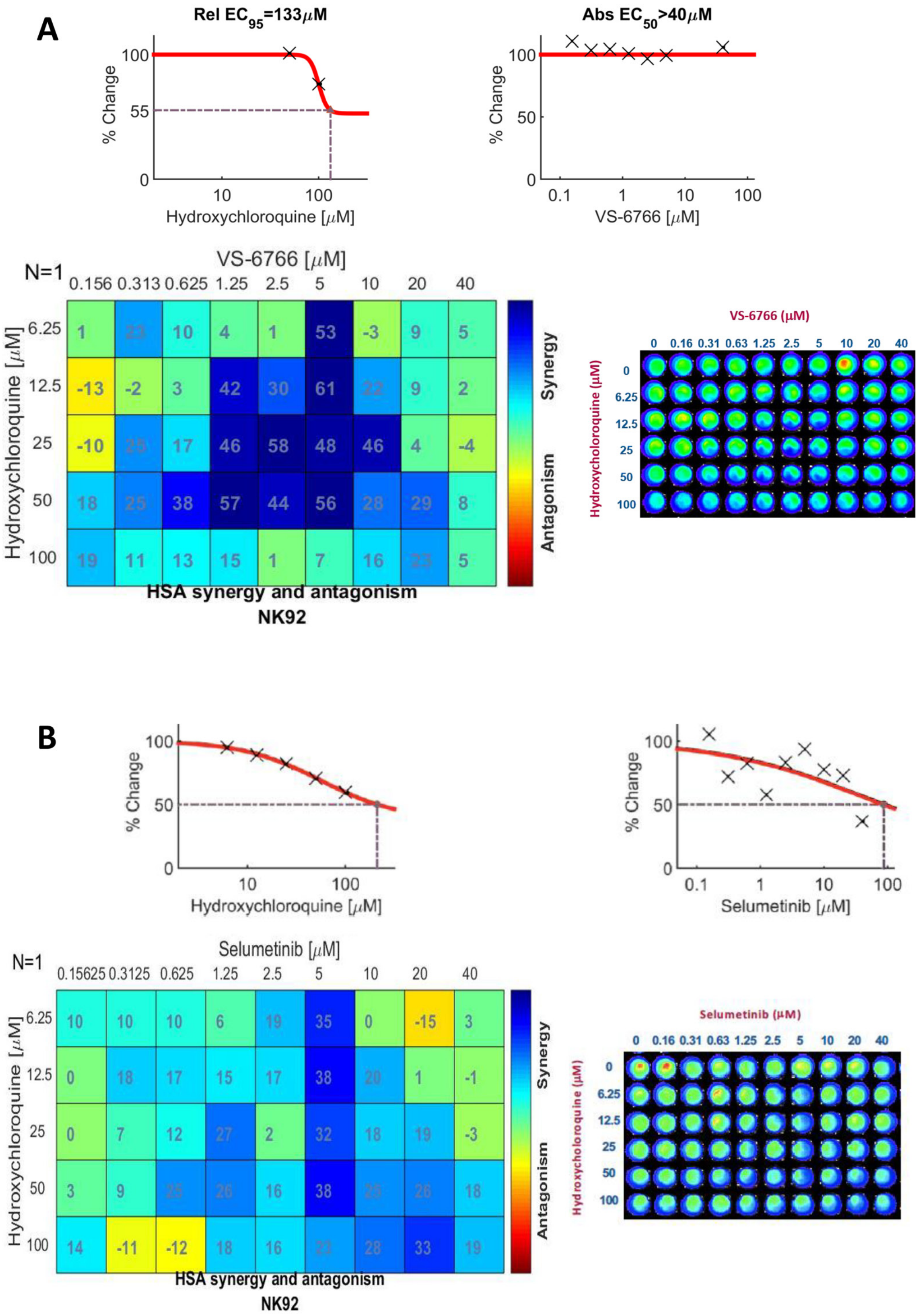

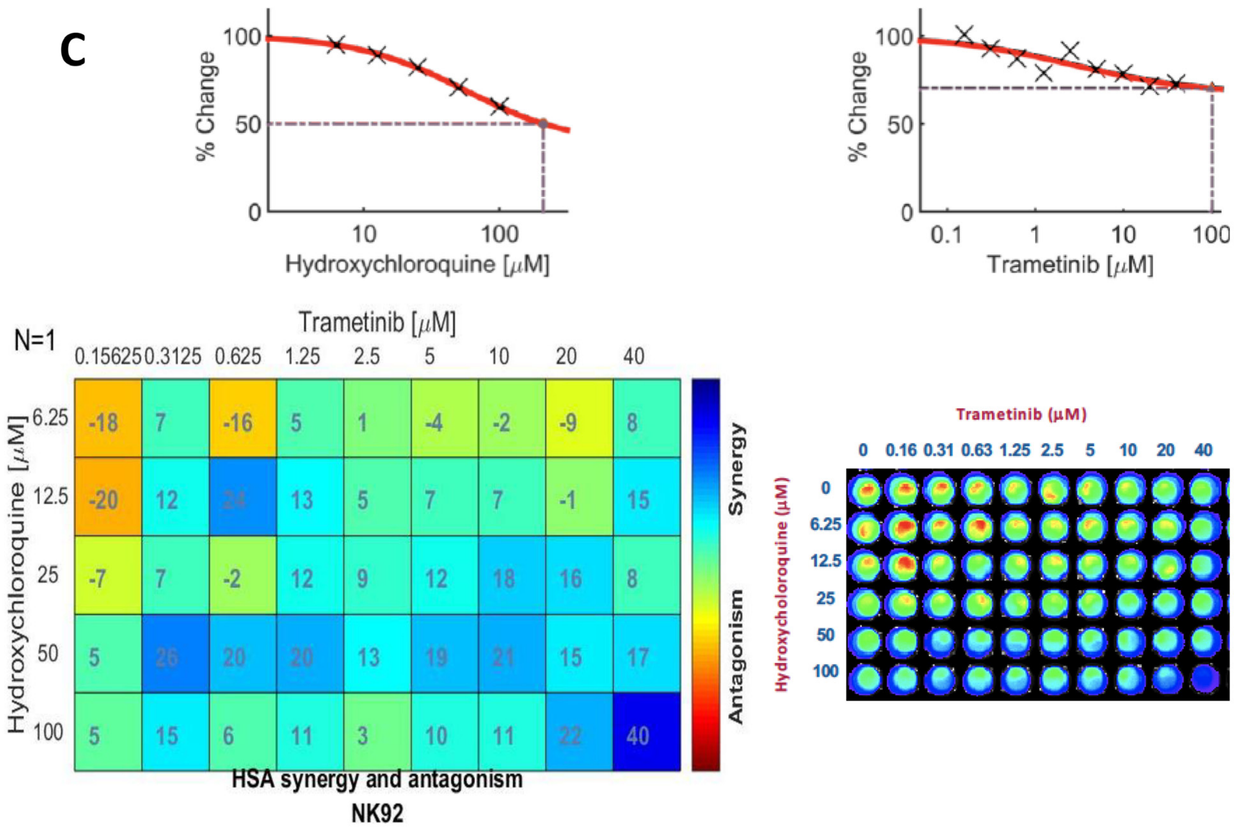


**Supplementary Figure 8:** Combinational effect of Hydroxychloroquine and MEK inhibitors VS-6766 (**A**), Selumetinib (**B**) and Trametinib (**C**) on cell viability in TALL-104 cells. TALL-104 T cells were treated as indicated for 72 hours and CellTiter-Glo was added to acquire cell viability images with the Xenogen IVIS system. Combenefit was used to plot single agent dose response and synergy distribution matrix.

A

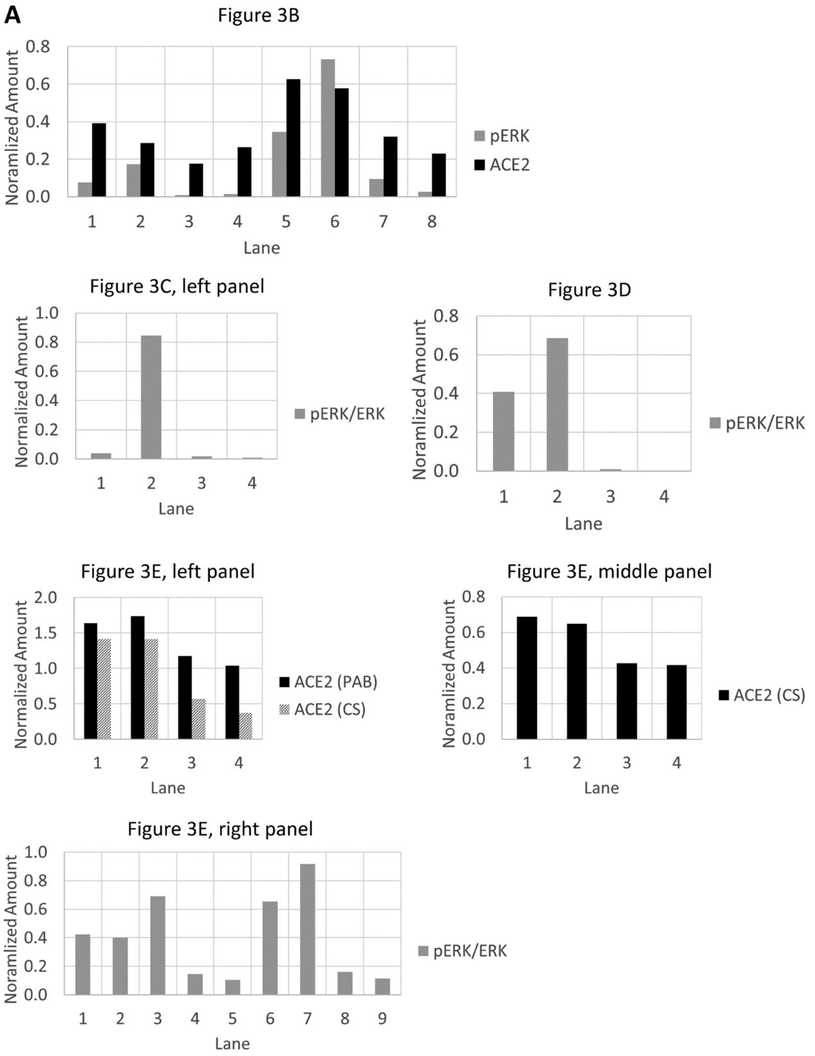
Figure 3B
Noramlized Amount
Lane
pERK
ACE2
Figure 3C, left panel
Normalized Amount
Lane
pERK/ERK
Figure 3D
Noramlized Amount
Lane
pERK/ERK
Figure 3E, left panel
Normalized Amount
Lane
ACE2 (PAB)
ACE2 (CS)
Figure 3E, middle panel
Noramlized Amount
Lane
ACE2 (CS)
Figure 3E, right panel
Noramlized Amount
Lane
pERK/ERK

B

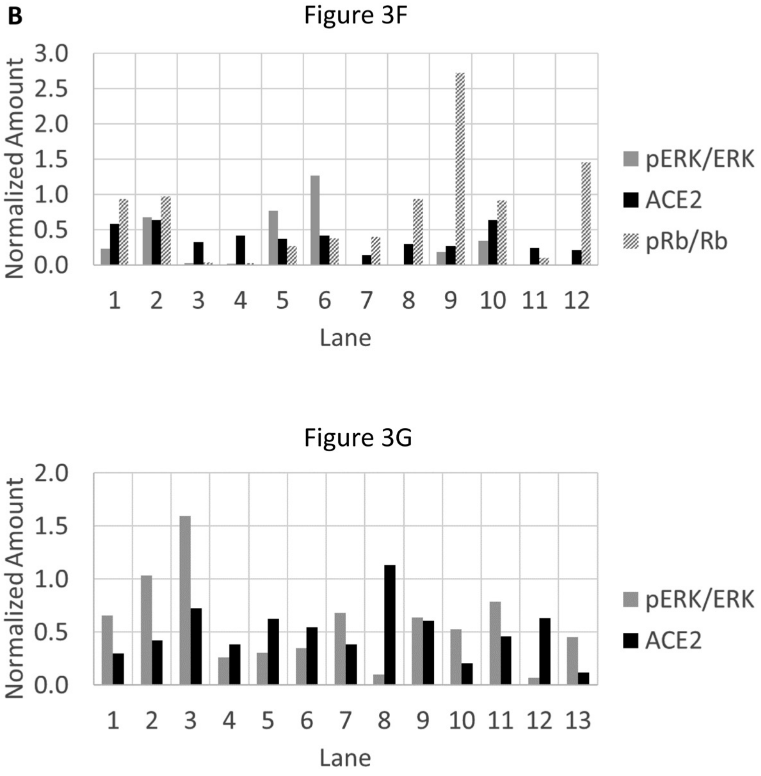
Figure 3F
Normalized Amount
Lane
pERK/ERK
ACE2
pRb/Rb
Figure 3G
Normalized Amount
Lane
pERK/ERK
ACE2

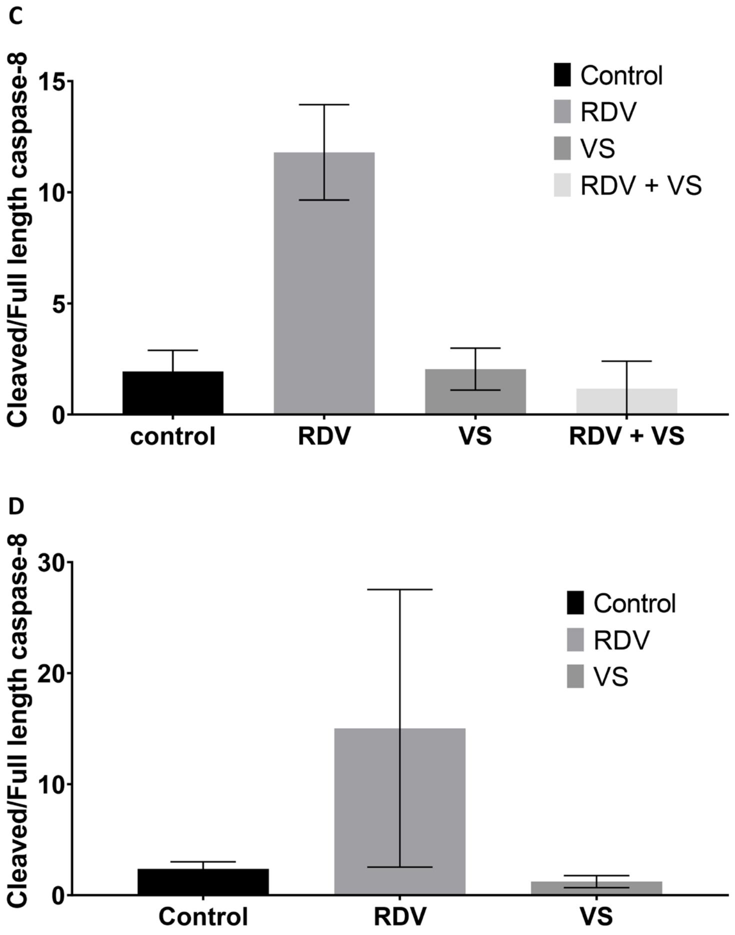


**Supplementary Figure 9: Quantification of Western blots.** ImageJ was used to quantify protein amounts from western blots shown in Figure 3B–3G (**A**–**B**). Cleaved and full-length caspase-8 were quantified from Figure 5G (**C**) and Figure 5F (**D**) as well as from additional experimental replicates. Standard deviation was calculated from 3–4 (C) or 2 (D) replicates.

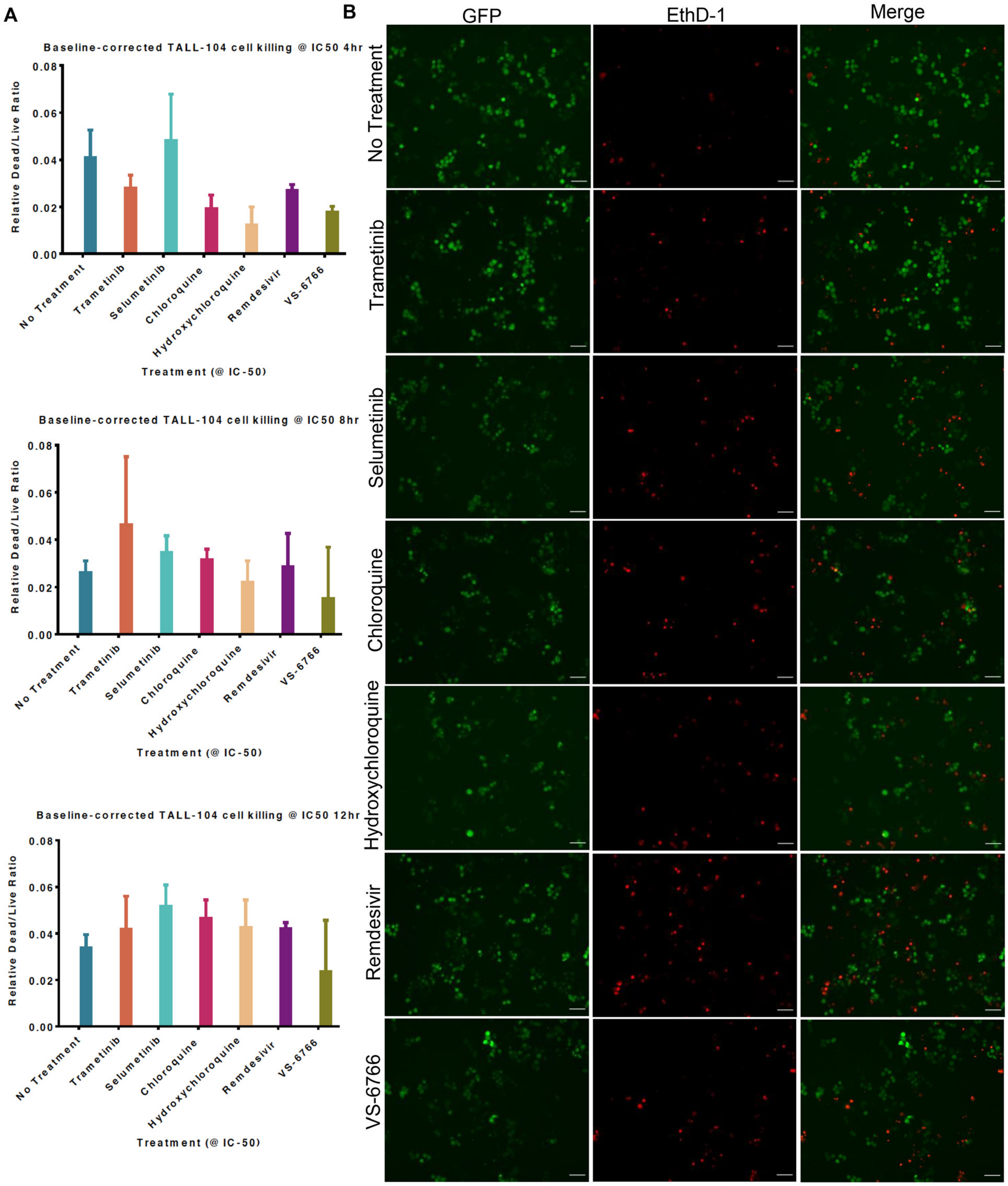
A
Baseline-corrected TALL-104 cell killing @ IC50 4hr
Relative Dead/Live Ratio
0.00
0.02
0.04
0.06
0.08
No Treatment
Trametinib
Selumetinib
Chloroquine
Hydroxychloroquine
Remdesivir
VS-6766
Treatment (@ IC-50)
Baseline-corrected TALL-104 cell killing @ IC50 8hr
Relative Dead/Live Ratio
0.00
0.02
0.04
0.06
0.08
No Treatment
Trametinib
Selumetinib
Chloroquine
Hydroxychloroquine
Remdesivir
VS-6766
Treatment (@ IC-50)
Baseline-corrected TALL-104 cell killing @ IC50 12hr
Relative Dead/Live Ratio
0.00
0.02
0.04
0.06
0.08
No Treatment
Trametinib
Selumetinib
Chloroquine
Hydroxychloroquine
Remdesivir
VS-6766
Treatment (@ IC-50)
B
GFP
EthD-1
Merge
No Treatment
Trametinib
Selumetinib
Chloroquine
Hydroxychloroquine
Remdesivir
VS-6766

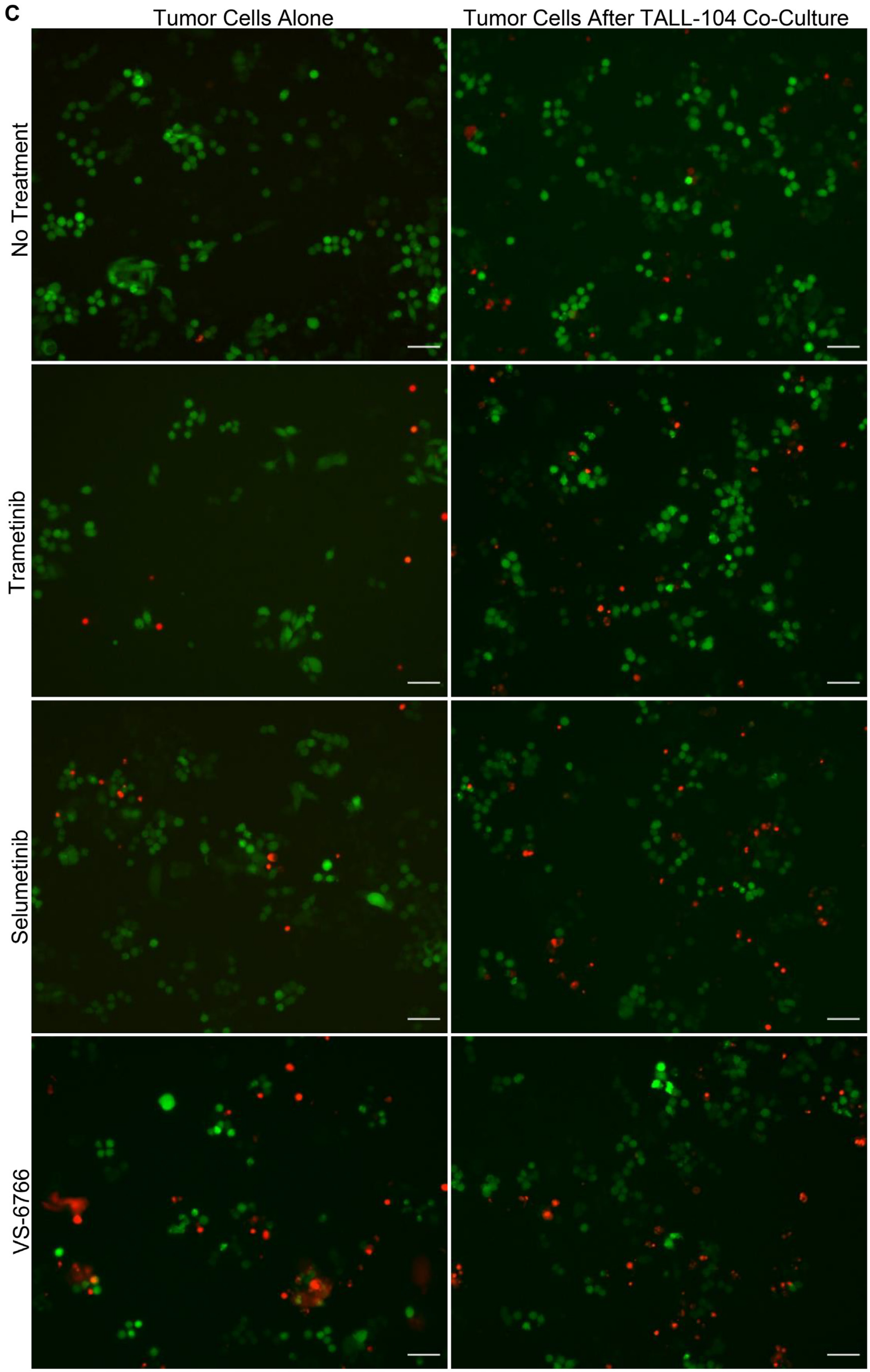
C
Tumor Cells Alone
Tumor Cells After TALL-104 Co-Culture
No Treatment
Trametinib
Selumetinib
VS-6766

D

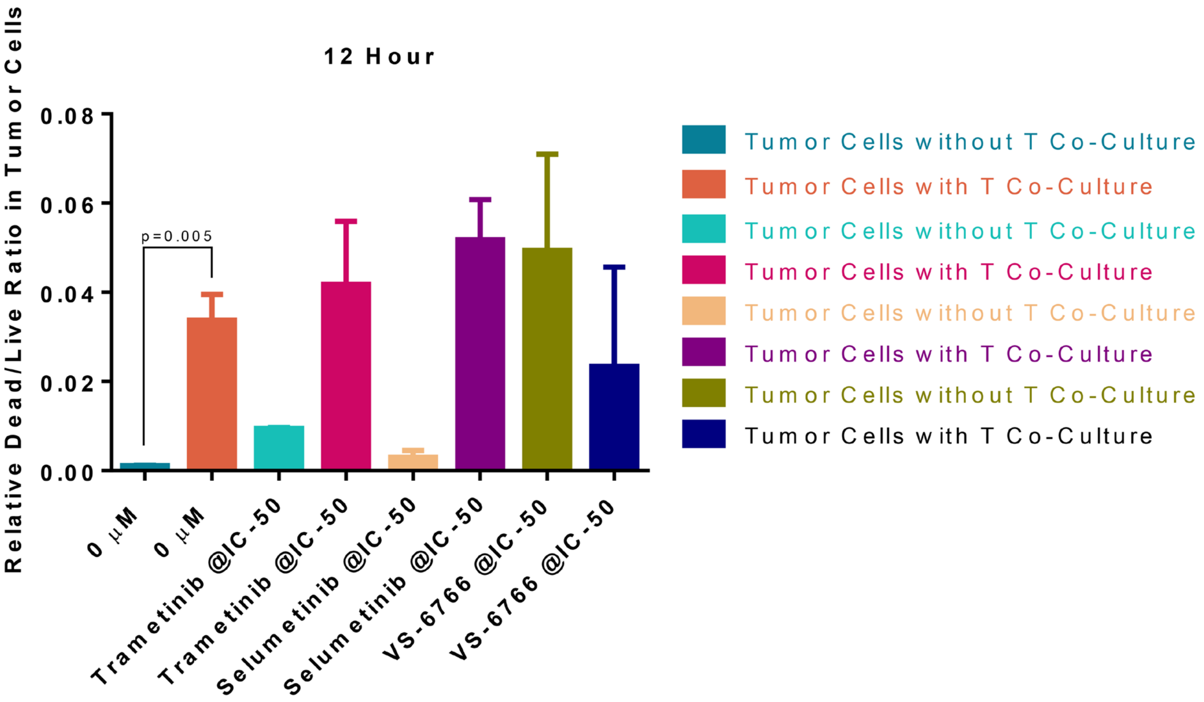


**Supplementary Figure 10: No effect observed on T cell activity by MEK inhibitor treatment at IC-50 doses.** Green fluorescent SW480 tumor cells were co-cultured with TALL-104 T cells at a 1:1 effector target cell ratio (E:T) for indicated timepoints and imaged. Cells were treated with indicated drug at IC-50 doses. (**A**) Quantification of dead/live ratio after 4, 8 and 12 hours of treatment by drugs as indicated. *P* values are displayed on graph and were calculated using unpaired *t* tests. (**B**) Fluorescent microscopy of GFP+ SW480 tumor cells before and after indicated treatment conditions. Ethidium homodimer was used to visualize dead cells. 10 magnification. Scale bars indicate 100 µM. (**C**) Images showing GFP+ tumor target cell cytotoxic effects of MEK inhibitors alone or in addition to TALL-104 cells. Ethidium homodimer was used to visualize dead cells. (**D**) Quantification of tumor target cell cytotoxic effects of MEK inhibitors alone or in addition to TALL-104 cells. *P* values are displayed on graph and were calculated using unpaired *t* tests.

**A**

| SW480 | |
|---|---|
| **Drug** | **IC50 (μM)** |
| Hydroxychloroquine | 26.92 |
| Chloroquine | 28.18 |
| Trametinib | 0.0011 |
| Selumetinib | 4.32 |
| Remdesivir | 11.22 |
| VS-6766 | 81.28 |

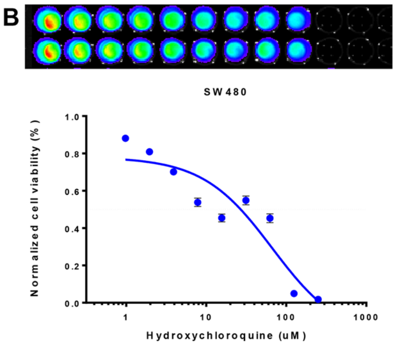


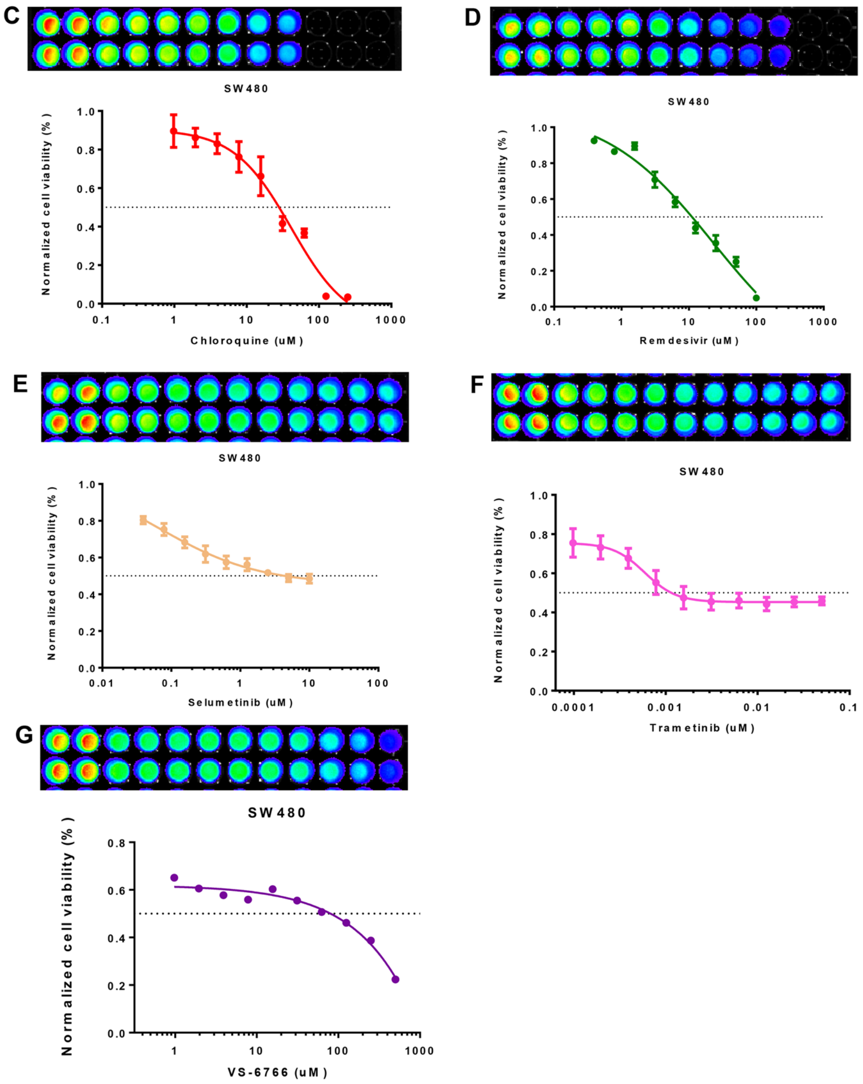


**Supplementary Figure 11:** IC-50 values for SW480 cell line (**A**) as determined by cell viability assays of SW480 treated with Hydroxychloroquine (**B**), Chloroquine (**C**), Remdesivir (**D**), and MEK inhibitors Selumetinib, Trametinib, and VS-6766 (**E**–**G**). SW480 tumor cells were treated as indicated for 72 hours and CellTiter-Glo was added to acquire cell viability images with the IVIS system. GraphPad Prism 6 was used to plot the dose-response curve using a non-linear regression curve fit and to calculate the IC50 values.

**A**

| NK-92 | |
|---|---|
| **Drug** | **IC50** |
| Chloroquine | 40.7 |
| Hydroxychloroquine | 42.7 |
| Remdesivir | 74.1 |
| Trametinib | >25 |
| Selumetinib | >25 |

**B**

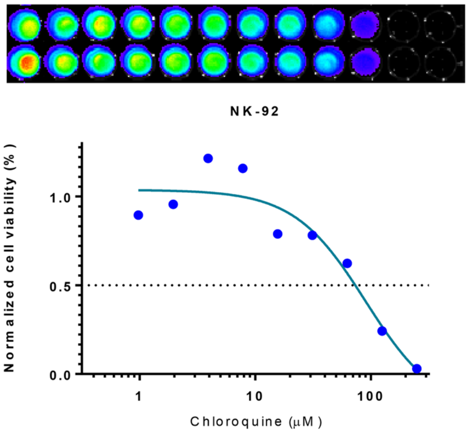


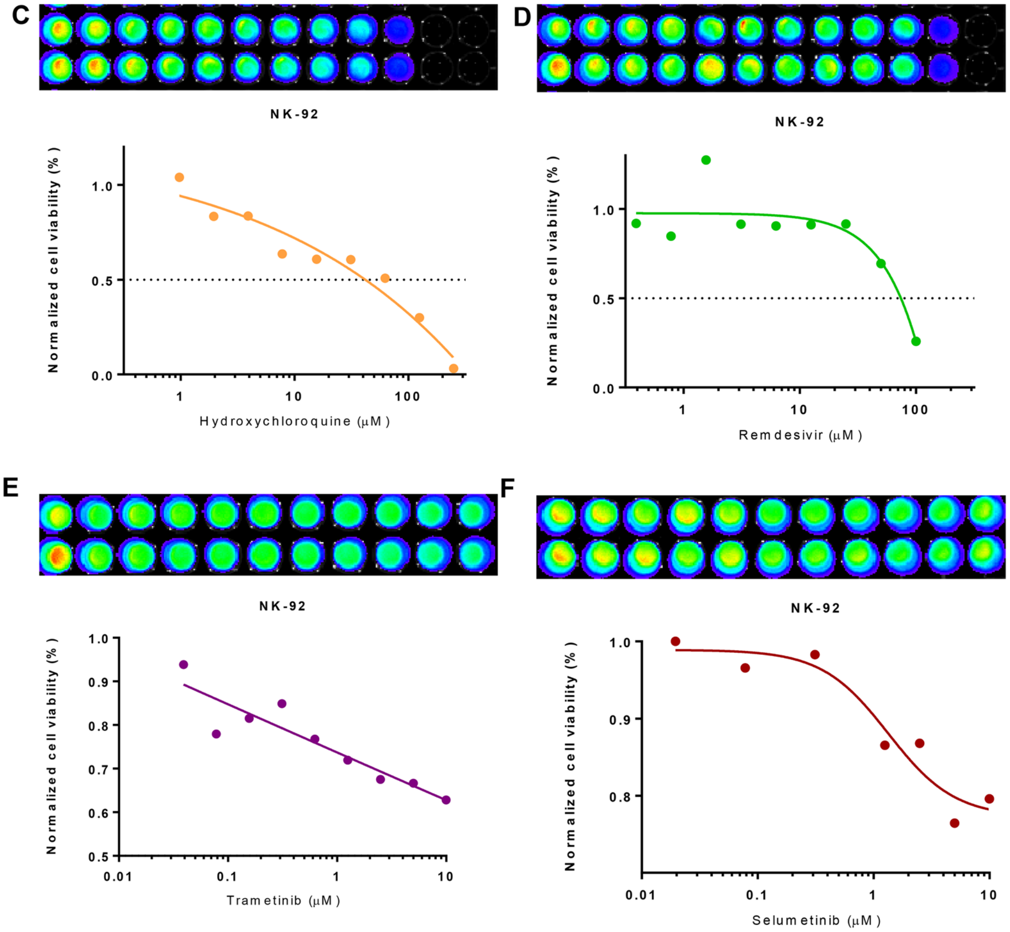


**Supplementary Figure 12:** IC-50 values for NK-92 cell line (**A**) as determined by cell viability assays of NK-92 cells treated with Chloroquine (**B**), Hydroxychloroquine (**C**), Remdesivir (**D**), and MEK inhibitors Trametinib (**E**) and Selumetinib (**F**). NK-92 natural killer cells were treated as indicated for 72 hours and CellTiter-Glo was added to acquire cell viability images with the IVIS system. GraphPad Prism 6 was used to plot the dose-response curve using a non-linear regression curve fit and to calculate the IC50 values.

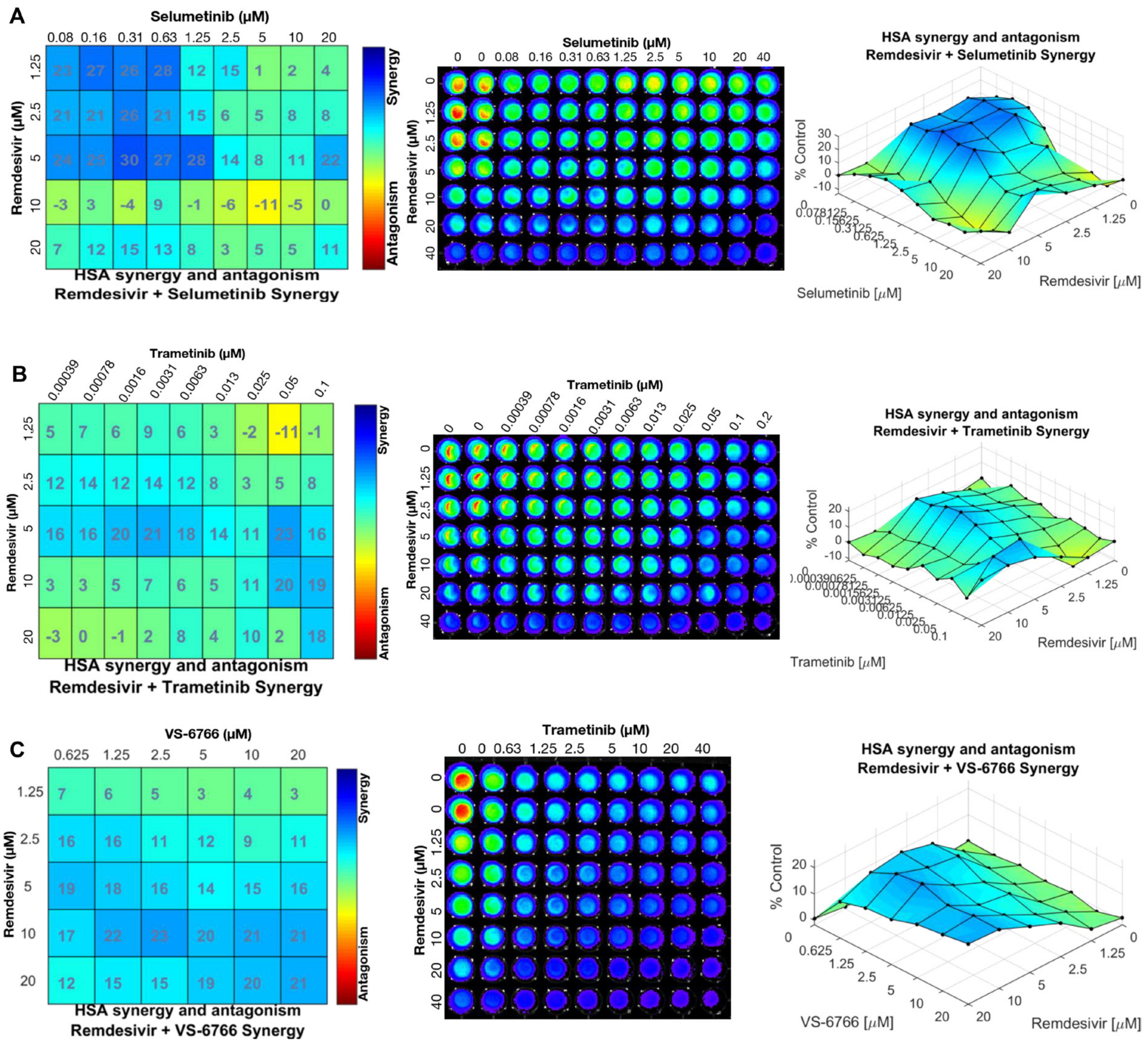


**Supplementary Figure 13:** Combinational effect of Remdesivir and MEK inhibitors Selumetinib (**A**), Trametinib (**B**) and VS-6766 (**C**) on cell viability in SW480 tumor cells. SW480 tumor cells were treated as indicated for 72 hours and CellTiter-Glo was added to acquire cell viability images with the Xenogen IVIS system. Combenefit was used to plot synergy distribution matrices and combination dose response surface models.

**Supplementary Table 1: Patient history.** See Supplementary Table 1

**Supplementary Table 2: Patient symptoms.** See Supplementary Table 2

**Supplementary Table 3: Interventions and medications in the emergency department.** See Supplementary Table 3

**Supplementary Table 4: COVID(+) patient analyte values (pg/mL).** See Supplementary Table 4
